# Prevalence of toxoplasmosis in pregnant women and vertical transmission of *Toxoplasma gondii* in patients from basic units of health from Gurupi, Tocantins, Brazil, from 2012 to 2014

**DOI:** 10.1371/journal.pone.0141700

**Published:** 2015-11-11

**Authors:** Marcos Gontijo da Silva, Marina Clare Vinaud, Ana Maria de Castro

**Affiliations:** 1 University Center UNIRG, Parasitology Laboratory, Av. Rio de Janeiro entre ruas 9 e 10, Centro, Gurupi—TO, Brazil; 2 Federal University of Goias (UFG), Tropical Medicine and Public Health Institute (IPTSP), Tropical Medicine and Public Health Post-Graduation Programme, Studies of the Host-Parasite Relationship Laboratory (LAERPH), Rua 235 esq. 1a. Av. s/n Setor Leste Universitário, CEP 74605–050, Goiânia, Brazil; University at Buffalo, UNITED STATES

## Abstract

**Introduction:**

Toxoplasmosis is a parasitary disease that presents high rates of gestational and congenital infection worldwide being therefore considered a public health problem and a neglected disease.

**Objective:**

To determine the prevalence of toxoplasmosis amongst pregnant women and vertical transmission of *Toxoplasma gondii* in their newborns attended in the Basic Units of Health (BUH) from the city of Gurupi, state of Tocantins, Brazil.

**Methods:**

A prevalence study was performed, including 487 pregnant women and their newborns attended in the BUH of the urban zone of the city of Gurupi, state of Tocantins, Brazil, during the period from February 2012 to February 2014. The selection of the pregnant women occurred by convenience. In the antenatal admission they were invited to participate in this study. Three samples of peripheral blood were collected for the detection of specific anti-*T*. *gondii* IgG, IgM and IgA through ELISA, for the polimerase chain reaction (PCR) and IgG avidity during pregnancy. When IgM antibodies were detected the fetal and newborn infection investigation took place. The newborn was investigated right after birth and after one year of age through serology and PCR to confirm/exclude the vertical transmission. The analyses were performed in the Studies of the Host-Parasite Relationship Laboratory (LAERPH, IPTSP-UFG), Goiania, state of Goias, Brazil. The results were inserted in a data bank in Epi-Info 3.3.2 statistic software in which the analysis was performed with p≤5%.

**Results:**

The toxoplasmosis infection was detected in 68.37% (333/487, CI95%: 64.62–72.86). The toxoplasmosis chronic infection prevalence was of 63.03% (307/487, CI95%: 58.74–67.32). The prevalence of maternal acute infection was of 5.33% (26/487; CI95%: 3.3–7.3) suspected by IgM antibodies detection in the peripheral blood. The prevalence of confirmed vertical transmission was of 28% (7/25; CI95%: 10.4–45.6).

**Conclusions:**

These results show an elevated prevalence of toxoplasmosis in pregnant women and vertical transmission of *T*. *gondii* in the city of Gurupi, state of Tocantins, Brazil.

## Introduction

Toxoplasmosis is a worldwide zoonosis caused by the protozoan *Toxoplasma gondii* (*T*. *gondii*) [1,2]. Toxoplasmosis is an important cause of miscarriage or adverse fetal effects such as neurological and ocular diseases and may also lead to late sequelae in the life of the infected newborn [3]. Toxoplasmosis prevalence varies among adult individuals depending on the studied population and on the age of the individuals [4]. These variations occur due to the difference of exposure to the main sources of infection which are: soil, water or food contaminated with feces from infected cats that contain *T*. *gondii* oocysts; or raw or uncooked meat that contain bradyzoit cysts [5]

The ubiquity of the infection source and the differential exposure of the individuals to it, due to cultural and hygienic habits, may explain why the prevalence of toxoplasmosis is extremely variable between countries and even within different regions of the same country [6–10]. One of the major causes of foodborne death in the United States is toxoplasmosis. It also represents an annual cost of illness around $3 billion in the same country [11]. The large variability of the toxoplasmosis prevalence described by the literature from studies performed in different regions characterizes the great regional variability of the incidence of this disease and also the specific characteristics of each studied population [6,12–14].

In spite of both maternal and congenital infections are frequently asymptomatic, the *T*. *gondii* infection is particularly severe when the primo infection occurs during pregnancy [15]. For instance, in developed countries such as USA, only in 10% of the infected pregnant women the disease is symptomatic with unspecific signs [16]. On the other hand, in developing countries such as Brazil, the prevalence of toxoplasmosis among pregnant women varies from 50 to 80% throughout the whole territory of this vast country [17].

The diagnosis of the gestational infection is based on serological tests that search for specific antibodies such as anti-*T*. *gondii* IgG, IgM and IgA. Usually these tests are used to confirm the infection due to their high sensitivity and specificity. It is important to highlight that serological tests for IgM may present persistently positive results for long periods; hence the IgG avidity test performed during the first trimester of pregnancy would help to determine whether it is a recent infection [18]. Therefore a positive IgM test result in a pregnant woman requires caution and further confirmation of acute infection. In fetuses and newborns the diagnosis of toxoplasmic infection is complex and performed through the union of serological and parasitological analysis. In newborns, anti-*T*. *gondii* IgM and IgA in any titer, anti-*T*. *gondii* IgG ascending titers or the detection of the parasitary DNA in the blood of the patient confirm the infection [3,18]. For the fetuses infection confirmation it is also recommended the amniocentesis to identify the parasite in the amniotic fluid by PCR or inoculation in mice if the maternal primary infection is diagnosed, if serologic testing cannot confirm nor exclude acute infection, or in the presence of abnormal ultrasound findings such as intracranial calcifications, microcephaly, hydrocephalus, ascites, hepatosplenomegaly, or severe intrauterine growth restriction [19]. This confirmation is essential for the prevention of a possible vertical transmission of *T*. *gondii* or the adequate treatment that may minimize the fetal sequelae [11]. For the newborn infection confirmation besides the serological tests from newborn and mother sera it is recommended to perform PCR on the cord blood, placenta and amniotic fluid collected at birth [20].

Most studies performed in Brazil aiming the determination of the toxoplasmosis prevalence were performed in Southeast, South and Mid-West regions [3,5–7,10,15,21,22] which present cultural and environmental differences when compared to North and Northeast ones [12,23]. Therefore the aim of this study was to determine the prevalence of gestational and congenital toxoplasmosis in pregnant women and their newborns attended in the Basic Units of Health from the urban zone of the city of Gurupi, state of Tocantins, North Region of Brazil.

## Materials and Methods

### Type of study, ethical aspects and population assessment

A cohort study was performed including 487 pregnant women attended at the 11 Basic Units of Health (BUH) from the urban and rural zone of the city of Gurupi, state of Tocantins, Brazil, during the period of February 2012 to February 2014. This study was approved by the Ethics in Research Committee from the University Center UNIRG, protocol number 394846.

The selection and inclusion of the pregnant women occurred during their admission at one of the BUH by convenience. The antenatal care for pregnant women occur in pre-established week days, therefore the researcher was present at the moment when the pregnant women were waiting for consultation. All pregnant women who freely presented themselves for the antenatal care consultation during the studied period and dwelled in the city of Gurupi were informed about the objective of this study and invited to participate. Aiming the non-disturbance of the routine of the medical service, the obtaining of the signed consent was performed after the antenatal care consultation in an appropriate room. All participants that accepted being part of this study provided a written consent to participate. When the pregnant women were underage the written consent was signed by their legal guardian.

The gestational age of first antenatal visit, number of antenatal visits and treatment follow up, when necessary, were extracted from the medical chart of the pregnant women after birth. The epidemiological data and risk factors associated to gestational toxoplasmosis in this studied group are appropriately explored by [24].

### Inclusion/exclusion criteria

The inclusion criteria were pregnancy confirmed by the clinician and by laboratory analysis with gestational age determined by the last period date and in accordance with the ultrasound analysis or determined by the ultrasound analysis performed before 20 weeks of gestational age. The ultrasound examination at pregnancy suspicion or confirmation is mandatory according to recommendations of the Health Ministry of Brazil [25]. The exclusion criteria were the non-consent to participate in the study, the uncertain gestational age, women who did not dwell in the city of Gurupi.

The criteria for the following of the toxoplasmosis in pregnant women and vertical transmission of *T*. *gondii* were positive results for IgM anti-*T*. *gondii* test, both IgM/IgG positive results or who presented seroconversion during pregnancy.

### Sample size

The parameters to estimate the sample size were: 50% of prevalence, reliability level (type I error) of 5% and precision level of 5%. A 5% of sample loss was estimated [26]. The 50% of prevalence was estimated based on the literature reports of population toxoplasmosis prevalence in Brazil [5]. A probabilistic sample of 487 pregnant women was selected.

### Laboratory techniques

As to preserve the internal validity of this study the same serological kit (same manufacturer) was used for the analysis of the blood sample from all pregnant women and their newborn. Also these analyses were always performed in the same laboratory by the same technician avoiding bias. The analyses were processed in the Studies of the Host-Parasite Relationship Laboratory (LAERPH) located at the Tropical Pathology and Public Health Institute of the Federal University of Goias, city of Goiania, state of Goias, Brazil. After the blood collection the samples were frozen and sent in sealed Styrofoam boxes to LAERPH. The maximal time interval for samples transportation was of 10 hours from the city of Gurupi to LAERPH in the city of Goiania.

One sample of 5 mL of peripheral blood was collected from each pregnant women at first, second and third gestational trimesters and at birth for the serological detection of IgA, IgM and IgG. The ELISA test for IgM and IgG antibodies detection was performed using the Imunotoxo Kit, Bioclin-Quibasa S/A^®^ from Brazil. The IgA antibodies were detected through Toxoplasma IgA ELISA kit, Immuno-biological Laboratories, Inc. (IBL-AMERICA, USA). To confirm the IgM positive results, a second blood sample (5mL) was immediately collected and the ELISA test was performed again accompanied by complementary tests such as specific anti-*T*. *gondii* IgG avidity and PCR to *T*. *gondii* detection.

Furthermore the blood samples from the newborns collected at birth were submitted to serological analysis to the identification of specific anti-*T*. *gondii* IgM, IgA, IgG antibodies, IgG avidity and PCR for *T*. *gondii* detection. One year after birth the children were submitted to another battery of the same tests to confirm the vertical transmission of *T*. *gondii* or to identify the transmission in the cases that it has not been identified previously. The vertical transmission was confirmed when there were positive results for IgM, IgA, low IgG avidity and/or persistence or increase in IgG antibody titers and also positive PCR results. The children with confirmed vertical transmission are being followed by a responsible clinician. Their clinical data and evolution are not described in this study.

The pregnant women that presented negative results to the serological analysis were followed aiming the vigilance of seroconversion during the three gestational trimesters. The pregnant women that presented an identification of acute infection were submitted to the same complementary tests.

At any moment when the detection of IgM antibodies occurred the pregnant women received treatment with spiramycin (500 mg/3 times a day) per 60 days prescribed by the physicians responsible for them. Also, during pregnancy, the congenital transmission was monitored by the medical team with ultrasound examinations. Amniocentesis is not available in BUH in Gurupi, therefore it was not performed in the pregnant women within this study. The responsible pediatrician did not prescribe treatment for any of the infected newborn during the period of this.

For the in house IgG avidity (Imunotoxo Kit, Bioclin-Quibasa S/A^®^ from Brazil) quantification two simultaneous plates were performed, A and B. The serum samples were diluted 1/200 and distributed into the two plates (100μL/cavity). After incubation during 30 minutes at 37°C, plate A was washed 5 times with the kit buffer and plate B was washed 3 times with a buffer supplemented with urea 6 M. Afterwards the two plates received the IgG conjugate as recommended by the manufacturer and incubated for 30 minutes at 37°C. After incubation and washing the chromogenic substrate was added and incubated for 10 minutes when the stop solution was added. The absorbance reading was performed by an automatic ELISA reader at 520nm (BIOTEC, LX800, Winooski, Vermont, EUA). The avidity index (AI) in percentage was calculated as the result of the absorbance reading from the plate washed with urea-buffer (U+) [Abs (U+)], divided by the absorbance reading from the plate washed with buffer (U-) [ABS (U-)], and multiplied by 100, as indicated by the formula: AI = Abs (U+)/ABS (U-) × 100 [27]. The results were expressed in avidity percentage and the parameters were as follows: low avidity–lower or equal to 30%; intermediary avidity–between 31 to 60%; high avidity–greater than 60% [28].

From the IgM positive blood samples (5mL) the PCR was performed as follows: the *T*. *gondii* DNA extraction from the total blood from the pregnant women and their newborn was performed using commercial kits for DNA extraction, BIOPUR from BIOMETRIX®. The reaction was performed in a total volume of 25μL containing 10mM TRIS HCl (pH 9.0), 3.5mM MgCl2, 0.2U Taq DNA Polymerase (Invitrogen), 0.5mM of each deoxynucleotide (dATP/ dTTP/ dGTP/ dCTP, Sigma Chemical Co., USA), 50 pmols of each reaction trigger (Invitrogen) and 2μL of DNA mold, to the reaction mix was added 40μL of mineral oil (Sigma Chemical Co. USA). The reactions were performed in a thermocycler Master Cycler Personal. The amplification program was constituted of an initial denaturation at 94°C (5min), 35 cycles of denaturation at 94°C (1 min), annealing at 62°C (1min) and extension at 72°C (1min) followed by the final extension at 72°C (10 min). A 126 pb fragment of the *T*. gondii B1 gene was targeted through the following pairs of primers: Toxo-B5 (5’-TGA AGA GAG GAA ACA GGT GGT CG-3’), Toxo-B6 (5’-CCG CCT CCT TCG TCC GTC GTA-3’). The sensitivity was of 92.9% and specificity of 100% [29]. Positive and negative previously tested samples were used as positive and negative controls. The products amplified by PCR with 100pb size were visualized by electrophoresis in 6% polyacrylamide gels revealed with silver stain [30].

### Statistical analysis

The obtained results were inserted in a specific data bank generated by the statistical software Epi-Info 3.3.2 which was used in the statistical analysis. Initially, frequency distribution tables were built for the categorical variables which allowed the calculation of tendency and dispersion measures. Afterwards contingency tables were prepared for the determination of association between independent variables and the serology results (dependent variable). The determination of the IgG avidity effect and the vertical transmission of *T*. *gondii* were performed through the multivariate logistic model which estimates the odds ratio (OR) with confidence interval of 95% between the formed subgroups and each variable. The significance level of 5% was adopted.

## Results

From February 2012 to February 2014, 550 pregnant women were invited to participate in this study. There were 49 refusals. 501 blood samples were collected but 14 (2.87%) were not processed due to the abandonment of this study from the pregnant women or due to the samples being considered unsuited for analysis. This occurred due to loss of the seal during transportation. However these losses did not compromise the intended sample size estimate. Blood samples from 487 pregnant women were considered fit for analysis which attended to the probabilistic statistical calculation of sample size.

All pregnant women participating in this study attended to at least one antenatal care consultation and 53% of them attended to six or more consultations as recommended by the Brazilian Health Ministry [31,32].

Regarding the beginning of the antenatal care, 195/487 (40.04%) of the pregnant women attended to the first consultation during the first trimester of gestational age, 290/487 (59.55%) during the second trimester and 2 (0.41%) during the third trimester as in Brazil the antenatal care is dependent of spontaneous seeking by the pregnant women. All 487 pregnant women were submitted to antenatal triage for toxoplasmosis through ELISA for the detection of IgG and IgM antibodies. The mean date of the first serology test was 18 weeks of gestational age.

There were 155 pregnant women with IgG and IgM negative serology. These women had their blood collected at the second and third trimesters of gestational age. All of them were submitted to three serological analyses. One case of seroconversion was detected which occurred during the second trimester of gestational age, therefore the number of toxoplasmosis negative pregnant women decreased to 154 (31.62%).

The toxoplasmosis infection was detected in 68.37% (333/487, CI95%: 64.62–72.86) considering all IgG and IgM positive results. Hence at the end of the study the prevalence of maternal chronic infection was of 63.03% (307/487) (CI95%: 58.74–67.32). The maternal acute infection was suspected in 24 cases during the first trimester of gestational age through IgM antibodies detection. All IgM positive antibodies were detected during the first trimester (24/26) with 2 IgA positive results. Also there was the exception of two pregnant women who presented IgM positive results at the second trimester of gestational age (2/26), form which one was confirmed by additional IgA antibodies detection and the other one through seroconversion (IgM) during the second trimester of gestational age. At the end there were 26 cases of possible acute infection diagnosed through the detection of IgM antibodies in a total of 5.33% (26/487, CI95%: 3.3%-7.3%) of the samples (Table 1).

**Table 1:** Specific anti-*Toxoplasma gondii* IgG, IgM and IgA antibodies prevalence in 487 pregnant women attended at Basic Units of Health in the city of Gurupi, state of Tocantins, Brazil, from February 2012 to February 2014.

Antibodies	n.	Prevalence	CI 95%
IgG (+); IgM (-); IgA (-)	307	63.04%	63.6%-72.2%
IgG (+); IgM (+); IgA (-)	22	4.51%	2.67%-6.36%
IgG (+); IgM (+); IgA (+)	4	0.82%	0.01%-1.62%
IgG (-); IgM (-); IgA (-)	154	31.62%	27.6%-36.0%
**Total**	**487**	**100%**	

(+) positive; (-) negative

The 26 blood samples from the acutely infected pregnant women were tested for IgG avidity antibodies. Low IgG avidity was found in five from seven pregnant women in which vertical transmission was confirmed. When evaluating the risk of vertical transmission related to the low IgG avidity an odds ratio of 42.5 (CI95%: 3.16–571.8) was found (Table 2). There was one miscarriage resulting in 25 mother/newborn pairs.

**Table 2:** Vertical transmission risk evaluation through the IgG avidity antibodies test in the 26 pregnant women detected with Specific anti-*Toxoplasma gondii* IgM during pregnancy in Basic Units of Health in the city of Gurupi, state of Tocantins, Brazil.

	With vertical transmission	Without vertical transmission	OR (CI 95%)	p
Low avidity	5/25	1/25	**42.5 (3.16–571.8)**	**0.003**
Intermediary avidity and High avidity	2/25	17/25		

It was possible to identify seven children with vertical transmission and one miscarriage. Paired blood samples were collected at birth, one from the mother and another from the newborn. Another paired blood sample collection was performed one year after birth. One newborn was not considered infected because the child did not receive medication during the studied period and presented a remarkable decrease in IgG titers after 12 months of age, high IgG avidity at birth and negative PCR results (patient 11, Table 3). The vertical transmission was confirmed in seven newborns (patients 1, 3, 4, 6, 18, 21, 26, Table 3), as follows: six newborns presented high titers of IgG antibodies (titers equal or superiors to the ones found in blood samples from the mother) at birth and that maintained high or increasing titers after 12 months of age (patients 1, 3, 4, 6, 18, 26, Table 3), and one newborn presented IgA antibodies detected from the blood sample collected at birth (patient 21, Table 3).

**Table 3:** Diagnostic evaluation in the 26 pregnant women detected with Specific anti-*Toxoplasma gondii* IgM during pregnancy attended in Basic Units of Health in the city of Gurupi, state of Tocantins, Brazil, from February 2012 to February 2014.

N.		ELISA SEROLOGY (5 BLOOD SAMPLES)												PCR (5 BLOOD SAMPLES)					Newborn prognosis
		1^st^		2^nd^		3^rd^		4^th^		5^th^									
		**IgG**	**IgM**	**IgG**	**IgM**	**IgG**	**IgM**	**IgG**	**IgM**	**IgG**	**IgM**	**IgA**	**AV**	**1**	**2**	**3**	**4**	**5**	
1	M	**P**	**P**	**PI**	**N**	**PI**	**P**	**P**	**P**	**P**	**N**	**N**	**L**	**N**	**N**	**P**	**N**	**N**	
	NB							**P**	**N**	**PI**	**N**	**N**	**L**				**N**	**P**	**Infected**
2	M	**P**	**P**	**PI**	**N**	**P**	**N**	**P**	**N**	**PD**	**N**	**N**	**H**	**N**	**N**	**N**	**N**	**N**	
	NB							**P**	**N**	**N**	**N**	**N**	**H**				**N**	**N**	**Non Infected**
3	M	**P**	**P**	**PI**	**N**	**P**	**P**	**P**	**P**	**P**	**N**	**N**	**L**	**N**	**N**	**P**	**N**	**N**	
	NB							**P**	**N**	**P**	**N**	**N**	**H**				**N**	**N**	**Infected**
4	M	**P**	**P**	**PI**	**N**	**P**	**P**	**P**	**P**	**P**	**N**	**N**	**H**	**N**	**N**	**P**	**P**	**P**	
	NB							**P**	**N**	**PI**	**P**	**N**	**L**				**N**	**N**	**Infected**
5	M	**P**	**P**	**PD**	**N**	**PI**	**N**	**P**	**N**	**P**	**N**	**N**	**H**	**N**	**N**	**N**	**N**	**N**	
	NB							**N**	**N**	**N**	**N**	**N**	**H**				**N**	**N**	**Non Infected**
6	M	**P**	**P**	**P**	**P**	**P**	**P**	**P**	**P**	**P**	**N**	**N**	**L**	**N**	**N**	**N**	**N**	**N**	
	NB							**P**	**N**	**P**	**N**	**N**	**L**				**N**	**N**	**Infected**
7	M	**P**	**P**	**PI**	**N**	**P**	**N**	**P**	**N**	**P**	**N**	**N**	**H**	**N**	**N**	**N**	**N**	**N**	
	NB							**P**	**N**	**N**	**N**	**N**	**H**				**N**	**N**	**Non Infected**
8	M	**P**	**N**	**PI**	**P**	**P**	**N**	**P**	**N**	**P**	**N**	**P**	**I**	**N**	**N**	**N**	**N**	**N**	
	NB							**P**	**N**	**N**	**N**	**N**	**H**				**N**	**N**	**Non Infected**
9	M	**P**	**P**	**PI**	**N**	**P**	**N**	**P**	**N**	**P**	**N**	**N**	**I**	**N**	**N**	**N**	**N**	**N**	
	NB							**P**	**N**	**N**	**N**	**N**	**H**				**N**	**N**	**Non Infected**
10	M	**P**	**P**	**PI**	**N**	**PI**	**N**	**P**	**N**	**P**	**N**	**N**	**L**	**N**	**N**	**N**	**N**	**N**	
	NB							**N**	**N**	**N**	**N**	**N**	**H**				**N**	**N**	**Non Infected**
11	M	**P**	**P**	**PI**	**P**	**P**	**P**	**P**	**P**	**PD**	**N**	**N**	**I**	**N**	**P**	**N**	**N**	**N**	
	NB							**P**	**N**	**PD**	**N**	**N**	**H**				**N**	**N**	**Non Infected**
12	M	**P**	**P**									**N**	**I**	**N**					**Misscarriage 1** ^**st**^ **trimester**
	NB																		
13	M	**P**	**P**	**P**	**N**	**PD**	**N**	**P**	**N**	**PD**	**N**	**N**	**L**	**N**	**N**	**N**	**N**	**N**	
	NB							**P**	**N**	**N**	**N**	**N**	**H**				**N**	**N**	**Non Infected**
14	M	**P**	**P**	**P**	**N**	**P**	**N**	**P**	**N**	**PD**	**N**	**N**	**I**	**N**	**N**	**N**	**N**	**N**	
	NB							**P**	**N**	**N**	**N**	**N**	**H**				**N**	**N**	**Non Infected**
15	M	**P**	**P**	**PD**	**N**	**PD**	**N**	**P**	**N**	**PD**	**N**	**P**	**H**	**N**	**N**	**N**	**N**	**N**	
	NB							**P**	**N**	**N**	**N**	**N**	**H**				**N**	**N**	**Non Infected**
16	M	**P**	**P**	**P**	**N**	**P**	**N**	**P**	**N**	**P**	**N**	**N**	**I**	**N**	**N**	**N**	**N**	**N**	
	NB							**P**	**N**	**N**	**N**	**N**	**H**				**N**	**N**	**Non Infected**
17	M	**P**	**P**	**PI**	**N**	**PI**	**N**	**P**	**N**	**PD**	**N**	**N**	**I**	**N**	**N**	**N**	**N**	**N**	
	NB							**P**	**N**	**N**	**N**	**N**	**H**				**N**	**N**	**Non Infected**
18	M	**N**	**N**	**P**	**P**	**PI**	**N**	**PI**	**N**	**PD**	**N**	**N**	**I**	**N**	**N**	**N**	**N**	**N**	
	NB							**P**	**N**	**P**	**N**	**N**	**H**				**N**	**N**	**Infected**
19	M	**P**	**P**	**PI**	**N**	**PI**	**N**	**P**	**N**	**P**	**N**	**P**	**I**	**N**	**N**	**N**	**N**	**N**	
	NB							**P**	**N**		**N**	**N**	**H**				**N**	**N**	**Non Infected**
20	M	**P**	**P**	**PI**	**N**	**PI**	**N**	**P**	**N**	**P**	**N**	**N**	**H**	**N**	**N**	**N**	**N**	**N**	
	NB							**P**	**N**	**N**	**N**	**N**	**H**				**N**	**N**	**Non Infected**
21	M	**P**	**P**	**PI**	**N**	**PI**	**N**	**P**	**N**	**P**	**N**	**P**	**L**	**N**	**N**	**N**	**N**	**N**	
	NB							**P**	**N**	**P**	**N**	**P**	**L**				**N**	**N**	**Infected**
22	M	**P**	**P**	**PI**	**N**	**PD**	**N**	**P**	**N**	**P**	**N**	**N**	**I**	**N**	**N**	**N**	**N**	**N**	
	NB							**P**	**N**	**N**	**N**	**N**	**H**				**N**	**N**	**Non Infected**
23	M	**P**	**P**	**PI**	**N**	**PD**	**N**	**P**	**N**	**P**	**N**	**N**	**H**	**N**	**N**	**N**	**N**	**N**	
	NB							**P**	**N**	**N**	**N**	**N**	**H**				**N**	**N**	**Non Infected**
24	M	**P**	**P**	**PI**	**N**	**PD**	**N**	**P**	**N**	**P**	**N**	**N**	**H**	**N**	**N**	**N**	**N**	**N**	
	NB							**P**	**N**	**N**	**N**	**N**	**H**				**N**	**N**	**Non Infected**
25	M	**P**	**P**	**PI**	**N**	**PD**	**N**	**P**	**N**	**P**	**N**	**N**	**I**	**P**	**N**	**N**	**N**	**N**	
	NB							**P**	**N**	**N**	**N**	**N**	**H**				**N**	**N**	**Non Infected**
26	M	**P**	**P**	**PI**	**N**	**PI**	**N**	**P**	**N**	**P**	**N**	**N**	**L**	**N**	**N**	**N**	**N**	**N**	
	NB							**P**	**N**	**PI**	**N**	**N**	**H**				**N**	**N**	**Infected**

**N. patient identification number, M = mother, NB = newborn, AV = IgGavidity; P** = Positive, N = Negative, **PI** = increase in titers, **PD** = decrease in titers, **L** = low avidity titers, **I** = intermediary avidity titers, **H** = high avidity titers. 1^st^–first collection of blood at first trimester of gestational age; 2^nd^–second blood collection at second trimester of gestational age; 3^rd^–third collection of blood samples at third trimester of gestational age; 4^th^–fourth collection of blood collection at birth from mother and newborn; 5^th^–fifth collection of blood samples one year after birth from mother and child. The IgG avidity was performed in the first blood sample collection from the mother and at birth from the newborn.

In one of the newborns the confirmation occurred due to the increase in IgG antibodies titers and the detection of IgM antibodies after one year of age (patient 4, Table 3). There was another case in which the newborn had IgG antibodies titers higher than the ones from the mother both at birth and one year after (patient 1, Table 3). In this case it was possible to detect the parasitary DNA through PCR from peripheral blood at one year of age (Table 3). The PCR technique allowed the detection of *T*. *gondii* DNA in 19.23% (5/26, CI95%: 4.08–34.38) of the pregnant women. One of them was detected during the first trimester of gestational age, one at the second and three at the third trimester. One newborn (4%, 1/25, CI95%: -3.68–11.68) had *T*. *gondii* DNA detected from a blood sample collected at one year of age.

All 26 pregnant women that presented positive results for IgM antibodies received spiramycin treatment since the detection of the positive serology for a period of 60 days or until the IgM antibodies detection resulted negative. One of these women interrupted the treatment on her own account due to inconvenient side effects.

## Discussion

This is the first study aiming the identification of toxoplasmosis serology in pregnant women attended at BUH from the city of Gurupi and also in the state of Tocantins, North Region of Brazil. The importance of toxoplasmosis prevalence among pregnant women determination is due not only to define the adequate primary attention measures towards the susceptible women but also to establish the correct therapy aiming the reduction of the fetal sequelae [12,33–35]. The strikingly high prevalence of vertical transmission found in this study is related to risk factors described previously for this same population by Silva et al. [24] which are age, raw meat and *in natura* milk intake, schooling, work and poor hygienic habits during the meal preparation.

Women health care, especially during pregnancy, is of utmost importance in public health policies. The antenatal care provides the adequate moment to the implementation of prophylactic measures against maternal-fetal transmission of several diseases, including toxoplasmosis which presents high prevalence [36]. The first consultation of antenatal care should occur as precociously as possible [37], which is in discordance of the data found in this study in which the average first consultation period was of 18 weeks of gestational age.

Similarly to the results found in our study, other authors found low seroprevalence of anti-*T*. *gondii* antibodies ranging from 10 to 30% in other regions such as North America, South of Asia and North of Europe [4,24,38–41]. Moderate seroprevalence (30 to 50%) were found in other regions from Central and South Europe and high seroprevalence (>50%) were found in Latin America (Venezuela, Argentina and Equator) and in countries from Africa [4,33,40]. The IgG antibodies prevalence found in this study was of 63.03% (307/487) and considered elevated confirming the high prevalence described in Latin America and Africa [36,42–45].

In Brazil, the IgG antibodies prevalence among pregnant women is highly variable. Figueiro-Filho in the year 2005 [46] in the state of Mato Grosso do Sul found a prevalence of 91.6%, Avelino with 2004 [5] in the city of Goiania, state of Goiás, found 65% of prevalence, Reis with 2006 [47] in the city of Porto Alegre, state of Rio Grande do Sul, found 61.1% of prevalence, Areal and Miranda with 2008[48] in the city of Vitória, state of Espírito Santo, found 73.5% of prevalence and Porto in the year 2008 [49] in the city of Recife, state of Pernambuco, found 74.7% of prevalence.

The results of our study are similar to several epidemiological studies performed within pregnant women performed in the Brazilian territory. This fact may be explained to the low socioeconomic conditions of the general population that seek medical consultation in the public health system (SUS) in Brazil. Also due to the exposure to bradizoyt cysts found in raw or undercooked meat and in *in natura* milk and to oocysts that contaminate water, food and soil [50,51].

In our study the prevalence of IgM antibodies in pregnant women was of 5.3%. This data is quite superior as the ones reported in other studies in Brazil as described by Bittencourt [15] in the year 2012 in the cities of Palotina and Jesuitas, state of Parana, which found a prevalence of 1.1%. Porto [49] with 2005 in the city of Recife, state of Pernambuco, found a prevalence of 2.8%. On the other hand some studies reported IgM antibodies prevalence in accordance with our results such as Moura [6] in the year 2013 in the city of Niteroi, state of Rio de Janeiro, found a rate of 4.2% of prevalence and Avelino [52] in the year 2009 which reported a prevalence of 8.6% in the city of Goiania, state of Goias.

The high seroprevalence of specific anti-*T*. *gondii* antibodies may be related to low socioeconomic conditions and the low rates of schooling of the local population [53]. Some authors report that the risk of maternal-fetal transmission depends on three simultaneous factors: rate of tachyzoites parasitemia in maternal blood, placenta maturing index and competence of the immunological response against *T*. *gondii* from the mother which is classified as complete, deficient or absent [4]. This may be observed in our study due to the fact the tachyzoites parasitemia are indicative of acute infection in the pregnant woman which was confirmed by positive IgM and low IgG avidity which was observed in 5 cases of vertical transmission, one of them presented high IgG avidity and the other undetermined. The competence of the maternal immune system was followed by the decreasing of antibodies titers, this fact was observed in all the 19 pregnant women who did not transmit the parasite into their newborns. In those women was possible to observe a decrease and negativation of IgM titers.

Regarding the vertical transmission there were seven newborns infected among 487 pregnancies from infected mothers attended at BUH of the Gurupi city. These index represents a total of 14.37 cases in 1,000 live births (14.37/1,000) which is an index superior to most of the published studies from Brazil and worldwide [4]. One case of miscarriage occurred probably due to the intra-uterus toxoplasmic infection which could not be confirmed because the embryonary material was discarded. According to Torgerson and Mastroiacovo (2013) the estimated global incidence of congenital toxoplasmosis is of 190,100 annual cases (95% IC: 179,300–206,300), this corresponds to an incidence rate of approximately 1.5 cases in 1,000 live births [4]. The prevalence of congenital toxoplasmosis in Africa ranges from 2.0 to 2.4 cases in 1,000 live births, in North America the prevalence is 0.6/1,000, in Central America it is 1.8/1,000, in South America it is 3.4/1,000, in Europe it ranges from 0.5 to 1.6/1,000, in Asia it ranges from 0.8 to 1.3/1,000 and in Oceania it ranges from 0.6 to 1.1/1,000 live births [4,54].

There are some localities that present a high prevalence of congenital toxoplasmosis such as the vertical transmission found in this study. For instance, in Guatemala the prevalence of congenital toxoplasmosis is of 11 cases in 1,000 live births [55] and in Mexico the prevalence is of 18/1,000 live births [56].

In Brazil the reported prevalence range from 0.3 cases in 1000 live births to 34/1000 live births [57] (Table 4).

**Table 4:** Prevalence of congenital toxoplasmosis in different regions of Brazil.

City/ State/ Region	Prevalence	Sample size	Author
Goiânia /Goiás / Mid-West Region	34/1,000 live births	522	Avelino et al. 2009 [52]
Goiânia / Goiás/ Mid-West Region	6.0/1,000 live births	1,514	Rodrigues et al. 2014 [3]
Porto Alegre/ Rio Grande do Sul / South Region	0.9/1,000 live births	41,112	Varella et al. 2009 [58]
Porto Alegre/ Rio Grande do Sul / South Region	6/10,000	10,000	Lago et al. 2007 [21]
Porto Alegre/ Rio Grande do Sul / South Region	19.19/10,000	364,130	Camargo-Neto et al. 2004 [59]
Ribeirão Preto/ São Paulo / Southeast Region	3.3/10,000	15,182	Carvalheiro et al. 2005 [60]
Minas Gerais/ Southeast Region	12.99/10,000	146,307	Vasconcelos-Santos et al. 2009 [61]
Sergipe/ Northeast region	4/10,000	15,204	Inagaki et al. 2012 [62]
Belém/ Pará/North Region	1.0/1,000 live births	6,000	Bichara et al. 2012 [63]

In Brazil there are great difficulties in the adoption of prophylactic strategies through governmental programs due to local problems in the implementation of adequate measures [63,64]. This may happen in several levels such as: lack of health professionals who are committed to reality change; maneuvering of funding released by the Health Ministry to other programs of primary health attention; low quality of health education in public schools; low socioeconomic level of the general population attended in the public health services; and greater exposure to sources of contamination with *T*. *gondii* [58,65]. The risk factors to gestational toxoplasmosis infection are: intimate contact with pets such as cats and dogs; flee, cockroaches and rats infested environments; inadequate food storage; low educational level; and poor hygienic life conditions [22,33,36,60].

In our study all the 26 pregnant women who had an acute infection detected had their blood samples submitted to PCR analysis which resulted in the detection of the parasite in four samples. This may occur due to the fact that during pregnancy the parasite is circulating through the maternal organism for a prolonged period [64].

Another important finding in our study is that four newborns from the seven ones that presented vertical transmission also presented low IgG avidity antibodies. This fact has been reported as a risk of gestational toxoplasmosis [65, 66]. It is believed that this result might have happened due to the mixture of antibodies originated from the mother and the newborn in the blood collected at birth. Therefore this analysis may act as a strong indicative of fetal infection and may help in the diagnosis of vertical transmission.

Another peculiarity of this study was the protocol used to treat the acutely infected pregnant women. From the 26 pregnant women with acute gestational toxoplasmosis detected 24 were treated with spiramycin for a period of 60 days (500 mg/3 times a day). One pregnant woman interrupted the treatment due to a miscarriage during the first trimester of gestational age and another pregnant woman interrupted the treatment on her own account against medical advice due to side effects and, fortunately, her newborn did not present sequelae at birth. None of the pregnant women were treated for a period longer than 60 days. This procedure was adopted due to personal conviction of the physicians who were attending these women in the BUH in the city of Gurupi, state of Tocantins, Brazil.

In spite of the treatment it was possible to observe congenital infection in seven cases, one of the factors that may have contributed to this rate is the efficacy of the gestational treatment or the lack of treatment, when the pregnant woman does not comply with it. The acute toxoplasmosis treatment during pregnancy as a prophylactic measure to prevent vertical transmission is controversial. Nevertheless, the provided treatment was in disagreement with the recommended by the Health Ministry of Brazil which is treatment after laboratory confirmation of the infection with spiramycin until the 13^th^ week of gestational age followed by an alternation every three weeks with the triple scheme composed by sulphadiazine, folinic acid and pyrimethamine between the 14^th^ and 34^th^ weeks of gestational age followed by spiramycin until birth [23,25,66]. Overall the research concerning the therapeutic prevention of toxoplasmosis congenital infection is lacking and few studies approach the efficacy of drugs in this matter. Therefore more studies should be performed which would enable better prevention measures to be taken [67].

Probably the epidemiological profile of the pregnant women from Gurupi city is similar to the ones from the city of Goiania, which are geographically near to one another (600km), which has a reported high rate of congenital toxoplasmosis [52]. Also the treatment provided for the IgM positive pregnant women was not efficient in preventing the sequelae found in their newborn. Furthermore, Silva et al. (2014) reported the risk factors associated to toxoplasmosis seropositivity in pregnant women from Gurupi city, which are cultural habits such as ingestion of undercooked or raw meat and habit of eating in restaurants were the vegetables are not properly washed may contribute to the acute infection during pregnancy and therefore a high risk of congenital transmission [24]. To determine the factors that influence the high prevalence of vertical transmission of *T*. *gondii* in this population studies of the molecular epidemiology of the circulating strain of the parasite are necessary. These studies will improve the understanding of the complex host-parasite relationship in this region.

One of the limitations of this study was the convenience sampling of the pregnant women, which were invited to participate when they freely presented themselves at public units of health. All pregnant women from the Gurupi city that did not seek antenatal care in public units or that did not seek antenatal care at all did not have the chance to participate in this study. Also one of the BUH of this study is a reference center for risk pregnancies which could result in a bias of sampling in this study.

As a general recommendation we believe that independently of the type of implemented control program (antenatal or neonatal) it is of fundamental importance the standardization of antenatal health care for the prevention of vertical transmission of *T*. *gondii*.

## Conclusions

The prevalence of *T*. *gondii* infection in the studied population was considered elevated as well as the number of vertical transmission with laboratory confirmation. These data highlight the importance of the knowledge regarding the health of the population as to enable the implementation of specialized health care programs. Such programs should be directed to the local needs as to promote a decrease in the vertical transmission of toxoplasmosis.

## Supporting Information

S1 Fig Organization Chart. (TIF)

S1 Table Spreadsheet with global data. (XLSX)

S1 Text Cover Letter. (DOCX)
