# Supplementary figures and images for: Prevalence of toxoplasmosis in pregnant women and vertical transmission of Toxoplasma gondii in patients from basic units of health from Gurupi, Tocantins, Brazil, from 2012 to 2014
Source: PLoS One. 2015 Nov 11;10(11):e0141700. doi: 10.1371/journal.pone.0141700 (PMC4641701; doi:10.1371/journal.pone.0141700)

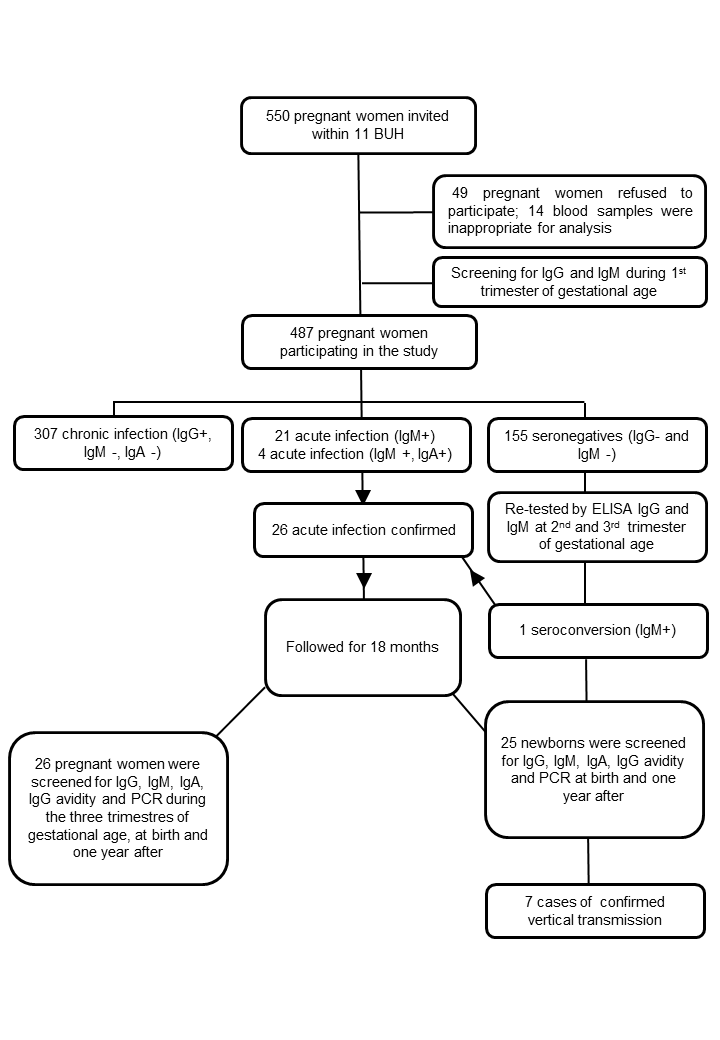

Supplement: S1 Fig — (TIF) [file pone.0141700.s001.tif]
