# Supplementary material for: Prevalence of toxoplasmosis in pregnant women and vertical transmission of Toxoplasma gondii in patients from basic units of health from Gurupi, Tocantins, Brazil, from 2012 to 2014
Source: PLoS One. 2015 Nov 11;10(11):e0141700. doi: 10.1371/journal.pone.0141700 (PMC4641701; doi:10.1371/journal.pone.0141700)
Supplement: S1 Text — (DOCX) [file pone.0141700.s003.docx]

Goiânia, 28^th^ of August, 2015.

To

*PLOS ONE* Editorial Board

Dear Editor,

We would like to submit the manuscript “**PREVALENCE OF TOXOPLASMOSIS IN PREGNANT WOMEN AND VERTICAL TRANSMISSION OF *TOXOPLASMA GONDII* IN PATIENTS FROM BASIC UNITS OF HEALTH FROM GURUPI, TOCANTINS, BRAZIL, FROM 2012 TO 2014.**” for publication as an original research paper in *PLOS ONE.* This study was performed with a group of pregnant women and their offspring who were attended by the public health system in the city of Gurupi, state of Tocantins, north region of Brazil.

This study is a result of a multicentric collaboration between the University Center (UNIRG) located in the city of Gurupi, state of Tocantins, and the Federal University of Goias (UFG) located in Goiania, state of Goias, both in Brazil. The authors would like to highlight the importance of this study due to its unpublished data from the state of Tocantins, north region of Brazil, regarding the toxoplasmosis prevalence in pregnant women and the congenital infection fulfilling an epidemiological gap about this disease in the mentioned region. The study presents substantial data regarding the prevalence of congenital toxoplasmosis which diverge from most of the published studies in this subject from other regions around the world.

As potential reviewers we suggest:

Dr. Glaucia Manzan Queiroz Andrade, professor, Universidade Federal de Minas Gerais, e-mail address: [qandrade.bh@terra.com.br](mailto:qandrade.bh@terra.com.br),

Dr. Ricardo Wagner Almeida Vitor, professor, Universidade Federal de Minas Gerais, e-mail address: [ricardovitor@icb.ufmg.br](mailto:ricardovitor@icb.ufmg.br),

Dr. Roberta Maia Castro Romanelli, professor, Universidade Federal de Minas Gerais, e-mail address: rmcromanelli@ig.com.br.

All authors had read the manuscript and accept responsibility for its contents as did the institution where it was performed. We also state that no portion of the study has been published or is under consideration for publication elsewhere. Furthermore, we declare no conflict of interests.

We are looking forward to your reply.

Yours faithfully,

_________________________________________________

Marcos Gontijo da Silva

_________________________________________________

Marina Clare Vinaud

_________________________________________________

Ana Maria de Castro
